# Prioritization of Lipid Metabolism Targets for the Diagnosis and Treatment of Cardiovascular Diseases

**DOI:** 10.34133/research.0618

**Published:** 2025-02-19

**Authors:** Zhihua Wang, Shuo Chen, Fanshun Zhang, Shamil Akhmedov, Jianping Weng, Suowen Xu

**Affiliations:** ^1^ Department of Endocrinology, Centre for Leading Medicine and Advanced Technologies of IHM, The First Affiliated Hospital of USTC, Division of Life Sciences and Medicine, University of Science and Technology of China, Hefei 230001, China.; ^2^ Institute of Endocrine and Metabolic Diseases, University of Science and Technology of China, Hefei 230001, China.; ^3^ Cardiology Research Institute, Tomsk National Research Medical Center, Russian Academy of Sciences, Tomsk 634012, Russia.; ^4^ Anhui Provincial Key Laboratory of Metabolic Health and Panvascular Diseases, Hefei 230001, China.

## Abstract

**Background:** Cardiovascular diseases (CVD) are a major global health issue strongly associated with altered lipid metabolism. However, lipid metabolism-related pharmacological targets remain limited, leaving the therapeutic challenge of residual lipid-associated cardiovascular risk. The purpose of this study is to identify potentially novel lipid metabolism-related genes by systematic genomic and phenomics analysis, with an aim to discovering potentially new therapeutic targets and diagnosis biomarkers for CVD. **Methods:** In this study, we conducted a comprehensive and multidimensional evaluation of 881 lipid metabolism-related genes. Using genome-wide association study (GWAS)-based mendelian randomization (MR) causal inference methods, we screened for genes causally linked to the occurrence and development of CVD. Further validation was performed through colocalization analysis in 2 independent cohorts. Then, we employed reverse screening using phenonome-wide association studies (PheWAS) and a drug target–drug association analysis. Finally, we integrated serum proteomic data to develop a machine learning model comprising 5 proteins for disease prediction. **Results:** Our initial screening yielded 54 genes causally linked to CVD. Colocalization analysis in validation cohorts prioritized this to 29 genes marked correlated with CVD. Comparison and interaction analysis identified 13 therapeutic targets with potential for treating CVD and its complications. A machine learning model incorporating 5 proteins for CVD prediction achieved a high accuracy of 96.1%, suggesting its potential as a diagnostic tool in clinical practice. **Conclusion:** This study comprehensively reveals the complex relationship between lipid metabolism regulatory targets and CVD. Our findings provide new insights into the pathogenesis of CVD and identify potential therapeutic targets and drugs for its treatment. Additionally, the machine learning model developed in this study offers a promising tool for the diagnosis and prediction of CVD, paving the way for future research and clinical applications.

## Introduction

Cardiovascular diseases (CVDs) remain the leading cause of morbidity and mortality globally, posing a substantial health burden on societies worldwide [1,2]. The intricate relationship between CVD and lipid metabolism has been well documented, with numerous studies highlighting the pivotal role of lipids in the pathogenesis of CVD [3,4]. However, the regulation of lipid metabolism is a complex process involving a vast array of genes, each with distinct and often overlapping functions [5,6]. Elucidating the causal relationships between these genes and CVD outcomes is essential for discovering potential therapeutic targets and devising efficient treatment approaches.

Despite significant advancements in our understanding of lipid metabolism and its association with CVD, a systematic and comprehensive evaluation of lipid metabolism-related genes in the context of CVD is lacking [7,8]. Traditional approaches have primarily focused on single-gene studies, which may overlook the complex interplay among multiple genes and their contributions to disease development [9,10]. Therefore, there is an urgent need for a multidimensional approach to systematically evaluate the role of lipid metabolism-related genes in CVD.

In this study, we aimed to fill this gap by conducting a comprehensive evaluation of 881 lipid metabolism-related genes using genome-wide association study (GWAS)-based causal inference methods. Our objective was to identify genes that are causally linked to the occurrence and development of CVD and to validate these findings in independent cohorts. Furthermore, we sought to identify potential therapeutic targets by employing reverse screening using phenome-wide association study (PheWAS) and a drug target–drug association database. Last, we integrated serum proteomic data to develop a machine learning model for the prediction of CVD, with the aim to providing a novel tool for disease diagnosis and prognosis. Our findings not only offer new insights into the pathogenesis of CVD but also pave the way for the development of targeted therapies and improved diagnostic strategies.

## Results

### Recognition of 25,115 valid QTLs linked to 609 lipid metabolism regulation genes

**Fig. 1.**
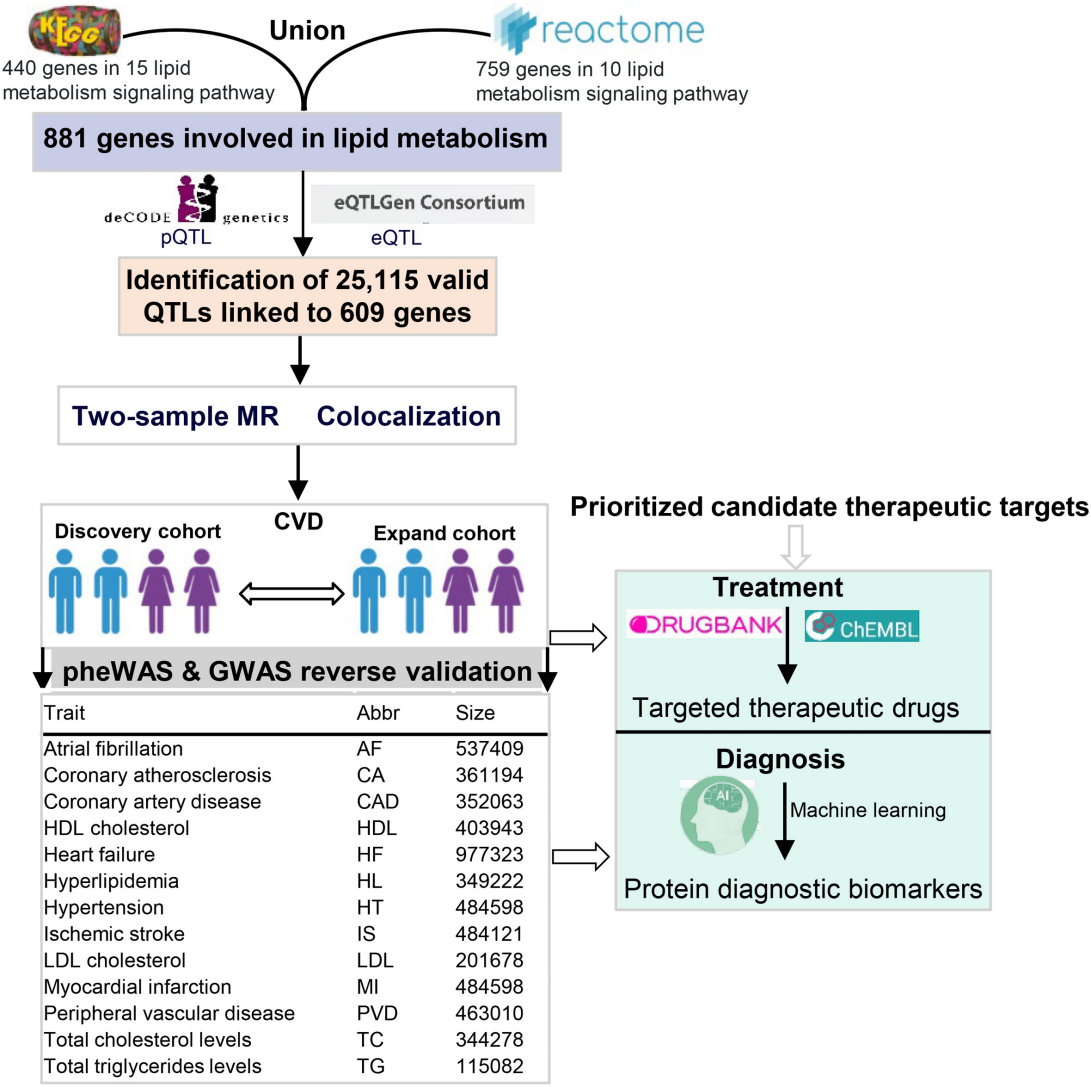
Study design for prioritization of lipid metabolism targets for the diagnosis and treatment of CVDs. Our study encompasses 2 primary components: Initially, we assign precedence to potential diagnostic and therapeutic biomarkers for CVD within lipid metabolism-regulating targets, employing techniques including GWAS analysis, MR analysis, colocalization analysis, and PheWAS analysis. Subsequently, utilizing drug–target interaction databases and serum proteomic datasets, we pinpoint probable targeted therapeutic agents and predictive diagnostic biomarkers for CVD based on these prioritized targets.

In this study, we aimed to identify a comprehensive set of genes involved in lipid metabolism regulation. As depicted in Fig. 1, we conducted a systematic analysis utilizing multiple public database resources, with all data sources detailed in Table S1. Briefly, we compiled a list of 881 genes involved in 25 signaling pathways related to lipid metabolism from both the Kyoto Encyclopedia of Genes and Genomes (KEGG) [11] and Reactome databases [12] (Tables S2 to S4). With this comprehensive list of genes in hand, we sought to identify their corresponding quantitative trait loci (QTLs). QTLs are genetic loci that influence the phenotypic traits that are quantitatively measured, such as gene expression levels. To achieve this, we leveraged 2 large-scale databases: eQTLGen [13] and deCODE [14]. These databases contain extensive QTL data derived from GWAS and other genetic analyses. By matching the 881 genes from our merged dataset with the QTLs in eQTLGen and deCODE, we were able to identify a significant number of QTLs that correspond to our genes of interest. We applied a stringent *P* value threshold of 1.00 × 10^−5^ to ensure the robustness of our findings, as this threshold is commonly used in genetic association studies to filter out false positives. After rigorous filtering, we matched 25,115 QTLs to 609 of the 881 genes in our dataset (Table S5). These QTLs represent genetic variants that are likely to influence the expression levels of these genes and, consequently, may play a role in regulating lipid metabolism.

### Identification of 54 lipid metabolism regulation genes associated with CVD via MR and colocalization analysis

**Fig. 2.**
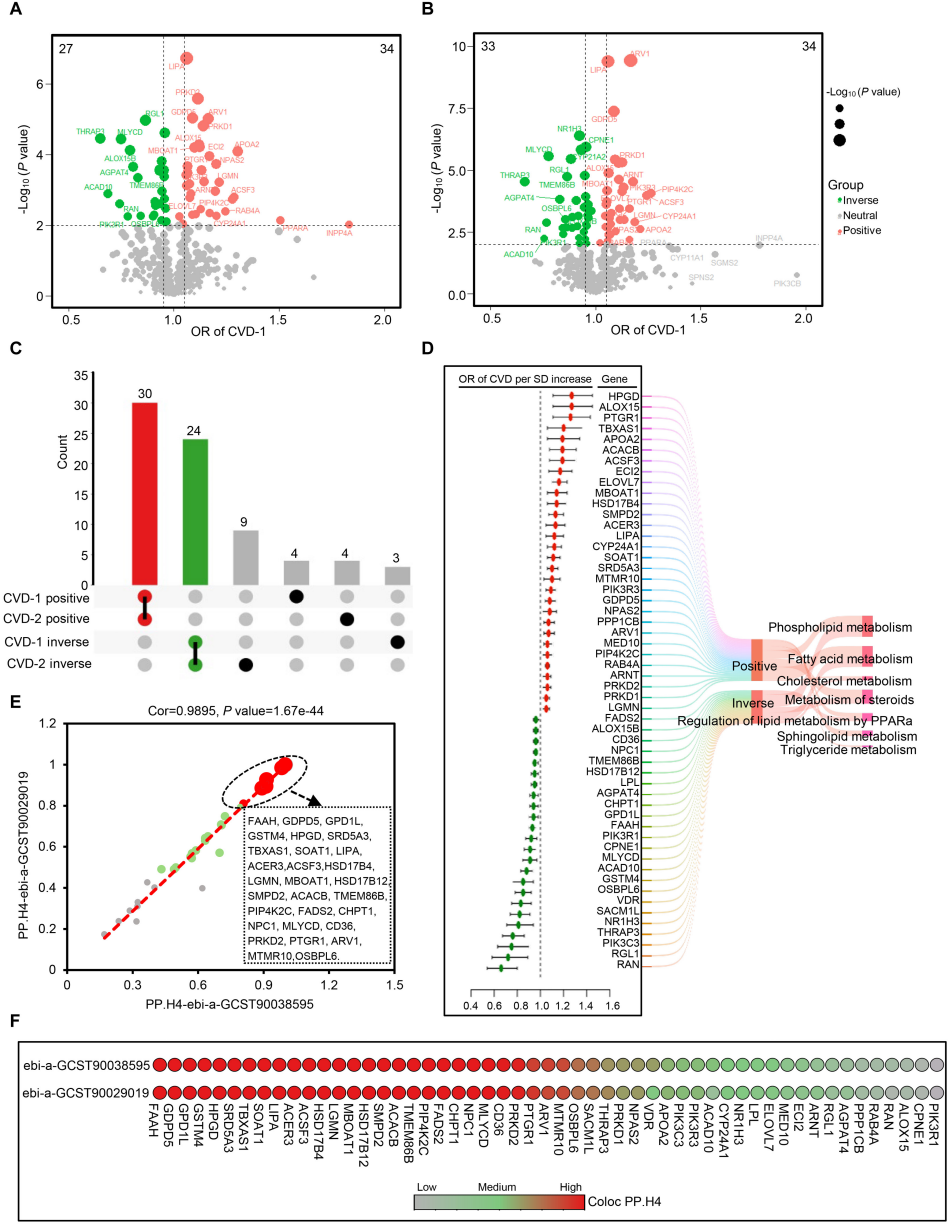
Summary of findings from MR and colorization studies on links between lipid metabolism regulatory targets and CVD risk. (A and B) The volcano plot illustrates the gene-wide MR analysis results for CVD, utilizing the discovery cohort ebi-a-GCST90086053 (*n* = 56,637) and finn-b-I9_CVD (*n* = 218,792). (C) The Upset plot visualizes MR analysis outcomes across various cohorts. (D) A Forest plot shows identified MR associations between lipid metabolism regulatory targets and CVD risk, based on the discovery cohort data. (E) Colocalization analysis results are presented using expanded cohort data (ebi-a-GCST90038595, *n* = 484,598; ebi-a-GCST90029019, *n* = 477,807). (F) A comparison of associations from the expanded cohort analysis is provided, based on ebi-a-GCST90038595 and ebi-a-GCST90029019 data.

Based on 25,115 QTLs associated with 609 genes regulating lipid metabolism, we conducted mendelian randomization (MR) analyses in 2 GWAS datasets with cohorts including CVD (Tables S6 and S7). The results revealed that 61 genes were identified to be associated with CVD in the ebi-a-GCST90086053 cohort analysis [odds ratio (OR) ≥ 1.05 or ≤ 0.95, and *P* ≤ 0.01; Fig. 2A]; similarly, 67 genes were found to be associated with CVD in the finn-b-I9_CVD cohort analysis (OR ≥ 1.05 or ≤ 0.95, and *P* ≤ 0.01; Fig. 2B). Among the MR analysis results from both cohorts, 54 genes (30 positively and 24 negatively correlated) were commonly identified (Fig. 2C and D). These 54 genes were primarily distributed across lipid metabolism signaling pathways such as Phospholipid metabolism, Fatty acid metabolism, Cholesterol metabolism, Metabolism of steroids, Regulation of lipid metabolism by PPARα, Sphingolipid metabolism, and Triglyceride metabolism (Fig. 2D), suggesting close associations between these pathways and the occurrence and development of CVD. Subsequently, we performed colocalization analyses with these 54 genes in 2 expanded GWAS cohorts for CVD (Tables S8 and S9). The results indicated that among the 54 gene–CVD associations, 29 had strong colocalization support with a PH4 value of >0.8, and 12 associations had medium colocalization support with 0.8 > PH4 > 0.5 (Fig. 2E and F). These findings not only confirm the impact of lipid metabolism-related signaling pathways on the occurrence and development of CVD but also further establish the causal relationship of specific lipid metabolism-regulating genes with CVD outcomes.

### Prioritization of 29 lipid metabolism targets for CVD therapeutic

To evaluate the “novelty” and “potential” of our priority targets, we developed a scoring system drawing inspiration from existing methodologies. This system comprises 6 criteria, with the total score being the sum of criteria met: (a) genes showing significance in CVD GWAS, with OR ≥ 1.05 or ≤ 0.95 and *P* ≤ 0.01; (b) genes identified as significant in CVD colocalization analysis, with PH4 value > 0.8; (c) genes prioritized through an exhaustive PubMed literature review; (d) genes exhibiting significance in CVD or associated complications; (e) phenotypes associated with CVD or complications, derived from gene-based PheWAS with *P* < 5 × 10^−8^; and (f) genes annotated as therapeutic targets in databases such as DrugBank [15], ChEMBL [16], and The Human Protein Atlas [17]. Targets meeting 4 or more criteria were classified as high potential, while those not meeting this threshold were considered relatively novel and understudied. Drug selection was based on their association with identified targets in DrugBank and ChEMBL, prioritizing direct target action and clinical stages: marketed drugs; phase III, II, I trials; and preclinical studies. In accordance with the aforementioned screening criteria, we ultimately prioritized 29 lipid metabolism targets for the treatment of CVD. Notably, 13 of these targets have previously been reported as drug targets and have been utilized in the treatment of other diseases (Table).

**Table.** Twenty-nine prioritized candidate therapeutic targets for CVD

Gene	CVD outcome	Colocalization	Duplication	MR-Egger inverse variance weighted	Drug development			
					Targets	Drug/compound	State	Disease
FADS2	Protection	✓	✓	✓	Known	α-Linolenic acid	Approved	Ligand/ASCVD
GSTM4	Protection	✓	✓	✓	Known	Glutathione	Approved	Liver failure/end-stage liver disease
HPGD	Risk	✓	✓	✓	Known	Alprostadil	Approved	Cardiovascular diseases/erectile dysfunction
SRD5A3	Risk	✓	✓	✓	Known	Finasteride	Approved	Prostatic hyperplasia; prostatic hypertrophy
NPC1	Protection	✓	✓	✓	Known	Ezetimibe	Approved	Cardiovascular diseases/hyperlipidemias
PIP4K2C	Risk	✓	✓	✓	Known	Fostamatinib	Phase 3	Rheumatoid arthritis/hemorrhage
PTGR1	Risk	✓	✓	✓	Known	Elafibranor	Phase 3	Liver cirrhosis/liver diseases
FAAH	Protection	✓	✓	✓	Known	JNJ-42165279	Phase 2	Phobic disorder/autism spectrum disorder
SOAT1	Risk	✓	✓	✓	Known	Nevanimibe	Phase 2	Cushing syndrome/congenital adrenal hyperplasia
LIPA	Risk	✓	✓	✓	Known	Afegostat	Phase 2	Gaucher disease
ACACB	Risk	✓	✓	✓	Known	Soraphen A	Experiment	Inhibitor
TBXAS1	Risk	✓	✓	✓	Known	Ridogrel	Experiment	Inhibitor
MLYCD	Protection	✓	✓	✓	Known	2-Carboxypropyl-coenzyme A	Experiment	/
GDPD5	Risk	✓	✓	✓	Unknown	/	/	/
GPD1L	Protection	✓	✓	✓	Unknown	/	/	/
ACER3	Risk	✓	✓	✓	Unknown	/	/	/
ACSF3	Risk	✓	✓	✓	Unknown	/	/	/
HSD17B4	Risk	✓	✓	✓	Unknown	/	/	/
LGMN	Risk	✓	✓	✓	Unknown	/	/	/
MBOAT1	Risk	✓	✓	✓	Unknown	/	/	/
HSD17B12	Protection	✓	✓	✓	Unknown	/	/	/
SMPD2	Risk	✓	✓	✓	Unknown	/	/	/
TMEM86B	Protection	✓	✓	✓	Unknown	/	/	/
CHPT1	Protection	✓	✓	✓	Unknown	/	/	/
CD36	Protection	✓	✓	✓	Unknown	/	/	/
PRKD2	Risk	✓	✓	✓	Unknown	/	/	/
ARV1	Risk	✓	✓	✓	Unknown	/	/	/
MTMR10	Risk	✓	✓	✓	Unknown	/	/	/
OSBPL6	Protection	✓	✓	✓	Unknown	/	/	/

### Reverse PheWAS validation of 29 prioritized genes for potential treatment of CVD

**Fig. 3.**
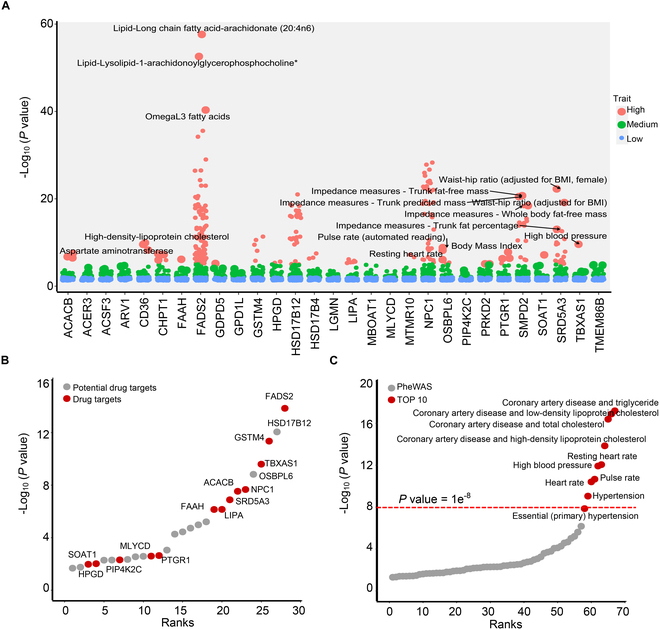
Phenome-level analysis for reverse identification of potential targets for CVD. (A) Manhattan plot for PheWAS of lipid metabolism regulation genes associated with CVD. (B) Molecular ranking chart Illustrating the contribution ranking of potential drug target genes. (C) Molecular ranking chart displaying the significance ranking of phenotypes associated with the genes.

Genes that regulate multiple related complications simultaneously often hold promise as therapeutic targets for diseases. Therefore, exploring comprehensive phenotype association analyses of genes represents a promising strategy for identifying drug targets. Herein, we conducted a phenotype scanning analysis by reviewing previous GWAS to uncover associations between identified genes and various traits. The results of the multi-trait phenotypic analysis of gene associations revealed that 29 genes were more or less associated with at least 3 to 5 complications of CVD (Fig. 3A). We ranked these genes based on their phenotypic contribution, and genes such as FADS2, HSD17B12, GSTM4, TBXAS1, OSBPL6, ACACB, NPC1, SRD5A3, FAAH, and LIPA made significant contributions to CVD-related phenotypes (Fig. 3B). When the CVD-related phenotypes associated with these genes were ranked by significance, the top 10 most significant phenotypes were coronary artery disease and triglyceride, coronary artery disease and low-density lipoprotein (LDL) cholesterol, coronary artery disease and total cholesterol, coronary artery disease and high-density lipoprotein (HDL) cholesterol, resting heart rate, high blood pressure, pulse rate, heart rate, and essential (primary) hypertension (Fig. 3C). Most of these traits are related to lipid metabolism and CVDs, suggesting that the aforementioned genes have potential as targets for regulating lipid metabolism homeostasis and treating CVD.

### Potential targeted therapy of 13 drug targets and their corresponding drugs for CVD

**Fig. 4.**
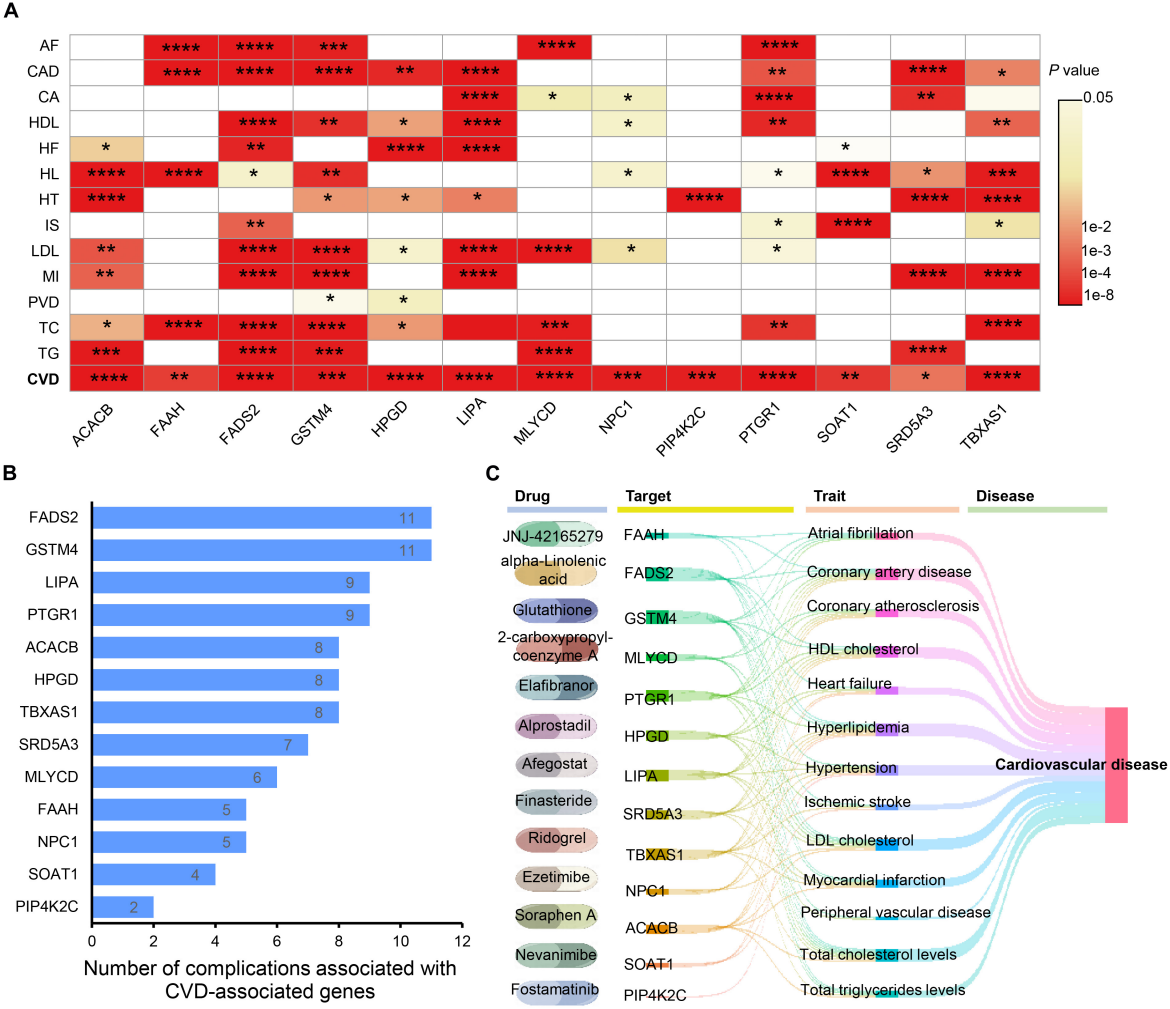
Analysis of associations and interactions involving 13 potential drug targets in CVD. (A) Results of MR analysis for 13 genes targeted by drugs and their associations with CVD and 13 related complications. (B) Summary of the influence of genes implicated in CVD on various complications. (C) Identified potential drug–target–trait–disease association network. AF, atrial fibrillation; CA, coronary atherosclerosis; CAD, coronary artery disease; HDL, HDL cholesterol; HF, heart failure; HL, hyperlipidemia; HT, hypertension; IS, ischemic stroke; LDL, LDL cholesterol; MI, myocardial infarction; PVD, peripheral vascular disease; TC, total cholesterol levels; TG, total triglyceride levels.

Although recognized as promising candidates for CVD therapy, the number of lipid metabolism-related targets directly applied in CVD treatment remains restricted. Here, we embarked on an extensive exploration of the relationship between 13 previously identified drug targets and CVD. Utilizing a sophisticated multi-phenotype MR analysis, we rigorously examined the associations between these 13 candidate genes and both CVD itself as well as 13 related complications (Fig. 4A). This analysis not only reinforced the significance of these genes in the context of CVD but also uncovered their widespread influence across multiple disease manifestations. Our findings were particularly striking for genes like FASD2, GSTM4, LIPA, PTGR1, ACACB, HPGD, TBXAS1, SRD5A3, MLYCD, and FAAH, which were implicated in at least 5 or more CVD-related complications (Fig. 4B). These genes emerged as key players in the complex etiology of CVD, suggesting that they may serve as promising targets for therapeutic intervention. Building on these insights, we conducted an exhaustive search for existing medications that target these identified potential causal proteins. By mining the DrugBank and ChEMBL databases, we were able to construct a comprehensive drug–target–triad–disease network (Fig. 4C). This network not only maps out the intricate relationships between drugs, targets, and disease manifestations but also provides a valuable resource for guiding precision treatment strategies in CVD. By leveraging this network, we can identify potential therapeutic candidates that specifically target the most relevant genes and pathways involved in CVD, thereby enhancing the efficacy and reducing the side effects of treatment.

### A machine learning model to predict CVD based on 5 proteins

In addition to exploring the potential therapeutic potential of candidate targets, we also attempt to investigate their potential disease prediction abilities. We derived the relative abundance of each target from serum proteome data, which included 30 subjects with CVD and an equal number of healthy controls (Table S10). Utilizing this dataset, we applied the well-established XGBoost machine learning technique to construct predictive models, and SHAP (SHapley Additive exPlanations) was utilized to interpret the analysis results.

**Fig. 5.**
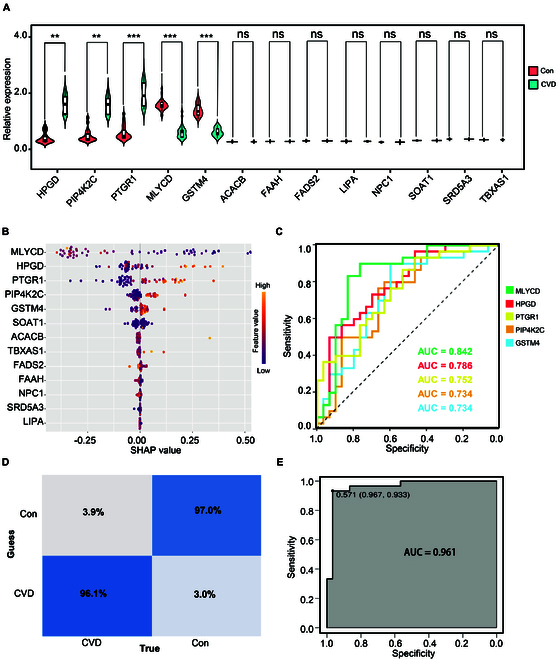
CVD prediction model using 5-protein machine learning. (A) Violin plot illustrating the serum proteome study of 13 drug target proteins in CVD patients versus healthy individuals. (B) Ranking of SHAP values predicted by machine learning. (C) ROC analysis plots and AUC values for five potential biomarkers associated with CVD. (D) Confusion matrix demonstrating the accuracy of the machine learning model in predicting CVD for the test and prediction sets. (E) ROC curve for CVD prediction based on the 5-protein model.

Proteomic data analysis revealed significant differential expression of 5 proteins, namely, HPGD, PIP4K2C, PTGR1, MLYCD, and GSTM4, between CVD patients and healthy individuals. Other proteins did not show significant differences, likely due to their low abundance as low-secretory proteins (Fig. 5A). The XGBoost machine learning predictions also indicated that MLYCD, HPGD, PTGR1, PIP4K2C, and GSTM4 contributed substantially to disease prediction (Fig. 5B). Their predictive accuracies for CVD were 0.842, 0.786, 0.752, 0.734, and 0.734, respectively (Fig. 5C). Subsequently, we established a new machine learning model based on these 5 proteins. The results demonstrated that this model achieved a predictive accuracy of 96.1% for CVD and 97% for non-CVD patients (Fig. 5D), with an overall precision of 0.961 (Fig. 5E). These findings highlight the promising application of our machine learning model based on a 5-protein biomarker panel for predicting CVD, thereby advancing the precise diagnosis of CVD.

## Discussion

Despite being widely recognized as promising potential therapeutic targets for CVD, the lipid metabolism-related targets reported for direct use in CVD treatment remain limited. Current therapeutic targets for lipid-related cardiovascular risk primarily include Lp(a), HDL-C, LDL-C, ABCA1, ANGPTL3, APOC3, CETP, PCSK9, and PPARα, among others [18–20]. However, most drug developments targeting these molecules are still in clinical or preclinical stages. Therefore, discovering new potential therapeutic targets and targeted drugs will significantly benefit the precision treatment of CVD. Based on the close correlation between lipid metabolism and CVD, this study aims to identify potential therapeutic targets for CVD from lipid metabolism-regulating genes. 

Multi-omics and multi-trait analysis often promise the discovery of therapeutic targets for many diseases [21–23]. In this study, we fully utilized various methods, including MR causal inference methods, colocalization analysis, PheWAS, and drug target–drug association, to systematically evaluate the associations between 881 lipid metabolism-related genes and GWAS data from multiple centers. We successfully identified 54 genes causally associated with CVD and selected 29 genes as candidate diagnostic and therapeutic targets. Additionally, we provided 13 therapeutic targets and their corresponding therapeutic drugs. The identification of these targets and drugs represents an important advancement in the search for effective CVD treatments, offering new avenues for clinical trials and therapeutic development.

Furthermore, although echocardiography, radionuclide angiography, computer tomography (CT), magnetic resonance imaging (MRI), and other techniques are available for heart disease examination, they are costly and unsuitable for dynamic monitoring [22,24]. Blood biochemical tests, however, provide important evidence for the diagnosis and treatment of heart diseases, especially coronary heart disease [24,25]. Given this, we also developed a machine learning model incorporating 5 proteins for CVD prediction. The model demonstrated a high accuracy of 96.1%, highlighting its potential utility as a diagnostic tool. The integration of serum proteomic data with machine learning techniques represents a novel approach in CVD prediction, enabling early identification of individuals at risk and timely interventions.

We acknowledge the inherent limitations of this study. Primarily, the MR and colocalization analyses relied on publicly available GWAS data from 4 distinct CVD cohorts, yielding results that represented their common intersections. Consequently, some genes that exhibited promising performance within individual cohorts, potentially representing effective populations, were excluded. Additionally, despite developing a machine learning model for CVD prediction using serum proteomic data incorporating 5 proteins, the scope of our detection was constrained by the limited sample availability. To address this, we are actively gathering more data to ascertain the model’s reliability. Last but not least, the causality of the identified 5 genes in CVD in our prediction model warrants further study in traditional animal models of CVD, including ApoE^−/−^ mice, LDLR^−/−^ mice, and LDLR^−/−^ hamsters [26,27].

## Conclusion

Our study provides a comprehensive evaluation of the complex relationship between lipid metabolism regulatory targets and CVD. By identifying causal genes, therapeutic targets, and developing a predictive machine learning model, we have contributed new insights into the pathogenesis of CVD and offered potential strategies for its prevention and treatment. The machine learning model, in particular, presents a promising tool for the diagnosis and prediction of CVD, with potential implications for personalized medicine and clinical decision-making. Future research should focus on further validating our findings in larger and more diverse ethnic populations, as well as exploring the functional mechanisms underlying the identified genetic associations. The directionality or causality of these identified genes in CVD needs to be validated in genetically modified animal models. Further studies are warranted to evaluate whether our machine learning model adds additional value to the diagnosis, prediction, and prognosis of CVD on top of LDL-based lipid risk and hypersensitive C-reactive protein (hs-CRP)-based inflammatory risk assessment. Last, clinical trials are also necessary to assess the effectiveness and safety of the potential treatments identified in CVD patients.

## Methods

### Study design

The description of the analytical workflow and research design is depicted in Fig. 1. Our analysis consists of 2 parts: First, we prioritize candidate diagnostic and therapeutic biomarkers for CVD among lipid metabolism-regulating targets using methods such as GWAS analysis, MR analysis, colocalization analysis, and PheWAS analysis. Second, based on these candidate targets, we identify potential targeted therapeutic drugs and predictive diagnostic biomarkers for CVD by integrating drug–target association databases and serum proteomic data. All data sources are shown in Table S1.

### Data sources of lipid metabolism targets

As depicted in Fig. 1, we embarked on this endeavor by collecting data from 2 reputable databases: KEGG and Reactome. Specifically, we gathered 440 genes associated with 15 lipid metabolism-related signaling pathways from the KEGG database (Table S2). Additionally, we obtained 759 genes linked to 10 lipid metabolism regulatory signaling pathways from the Reactome database (Table S3). To create a unified list of genes relevant to lipid metabolism regulation, we merged these 2 datasets, resulting in a total of 881 unique genes (Table S4). Subsequently, we identified corresponding potential quantitative trait locus (QTLs) for each gene in the eQTLGen and deCODE databases. Ultimately, we matched 25,115 QTLs (with *P* ≤ 1.00 × 10^−5^) that correspond to 609 genes (Table S5).

### Data sources for CVDs

In this study, we acquired data on the relationships between gene-related single-nucleotide polymorphisms (SNPs) and CVD from the integrative epidemiology unit (IEU) OpenGWAS project (accessible at https://gwas.mrcieu.ac.uk). This resource encompassed 4 cohorts: ebi-a-GCST90086053 (consisting of 56,637 samples) [28], finn-b-I9_CVD (comprising 218,792 samples) [29], ebi-a-GCST90038595 (484,598 samples) [30], and ebi-a-GCST90029019 (477,807 samples) [31]. For MR analysis, we designated the ebi-a-GCST90086053 cohort as the discovery set and the finn-b-I9_CVD cohort as the replication set. To bolster statistical power, we conducted a meta-analysis of the 2 GWAS datasets and subsequently performed colocalization analysis using the combined GWAS meta-analysis results derived from ebi-a-GCST90038595 and ebi-a-GCST90029019. The meta-analysis was executed utilizing RStudio (2024.04.2+764). Genetic variants exhibiting a significant association with CVD at a threshold of *P* < 5.00 × 10^−8^ in this meta-analysis and demonstrating minimal linkage disequilibrium (LD) (*R*^2^ < 0.001) were chosen as instrumental variables for CVD in the inverse MR analysis.

### Data sources for CVD complications

In this analysis, we included 13 CVD complications sourced from the IEU OpenGWAS project database. Specifically, these complications encompassed Atrial fibrillation (ID: ebi-a-GCST006061, *n* = 537,409) [32], Coronary atherosclerosis (ID: ukb-d-I9_CORATHER, *n* = 361,194) [33], Coronary artery disease (ID: ebi-a-GCST90013864, *n* = 352,063) [34], HDL cholesterol (ID: ieu-b-109, *n* = 403,943) [35], Heart failure (ID: ebi-a-GCST009541, *n* = 977,323)[36], Hyperlipidemia (ID: ebi-a-GCST90104006, *n* = 349,222) [37], Hypertension (ID: ebi-a-GCST90038604, *n* = 484,598) [30], Ischemic stroke (ID: ebi-a-GCST90018864, *n* = 484,121) [38], LDL cholesterol (ID: ieu-b-5089, *n* = 201,678) [39], Myocardial infarction (ID: ebi-a-GCST90038610, *n* = 484,598) [30], Peripheral vascular disease (ID: ukb-b-4929, *n* = 463,010) [33], Total cholesterol levels (ID: ebi-a-GCST90018974, *n* = 344,278) [38], and Total triglyceride levels (ID: ebi-a-GCST90092992, *n* = 115,082) [35]. Genetic variants that demonstrated a statistically significant association at a threshold of *P* < 5.00 × 10^−8^ in our meta-analysis and exhibited minimal LD (*R*^2^ < 0.001) for all aforementioned complications were selected as instrumental variables for the inverse MR analysis.

### MR analysis

In the context of MR analysis, SNPs associated with genes were designated as the exposure variables, whereas GWAS data for CVD derived from diverse cohorts were designated as the outcome variables. A total of 25,115 SNPs related to lipid metabolism-associated genes, with *P* < 1.00 × 10^−5^, were extracted from summary statistics (Table S5) and utilized as instrumental variables. Based on the European 1000 Genomes Project reference panel [40], LD clumping was conducted for each gene, applying an *r*^2^ cutoff of 0.01 and a 5,000-base pair window. This was followed by univariate 2-sample MR analyses. Phenotypes showing significant associations in at least 2 MR techniques, such as MR-Egger, inverse variance weighted (IVW), MR-PRESSO, and weighted median, were selected for further evaluation. Finally, volcano plots were generated using OR and *P* values to facilitate the identification of lipid metabolism-regulating genes with causal associations to CVD.

### Colocalization analysis

Bayesian colocalization analyses were conducted to evaluate the likelihood of 2 traits sharing a common causal variant, employing the “coloc” package (available at https://github.com/chr1swallace/coloc) [41] with default settings. As previously outlined, this approach computes the posterior probabilities for 5 hypotheses regarding the sharing of a single variant between 2 traits. In our study, we focused on assessing the posterior probabilities of hypothesis 3 (H3), which proposes that distinct variants associate the gene and CVD with the region, and hypothesis 4 (H4), which suggests that shared variants link both the gene and CVD to the region. We utilized both the coloc.abf and coloc.susie algorithms, and considered a gene to exhibit evidence of colocalization if the gene-based posterior probability for H4 exceeded 80%, as determined by at least one of the algorithms.

### PheWAS analysis

To explore the associations between candidate genes and other phenotypes, we conducted a phenotype scanning analysis by searching through previous GWAS to uncover links between the identified genes and various traits. This analysis utilized both the “phenoscanner” tool and the study by Kamat et al. [42]. An SNP was classified as pleiotropic if it met the following criteria: (a) the association achieved genome-wide significance (*P* < 5.00 × 10^−8^); (b) the GWAS was conducted in a population of European descent; and (c) the SNPs were associated with known risk factors of CVD, encompassing metabolic traits, proteins, and clinical characteristics. Ultimately, we ranked the genes based on their *P* values, prioritizing those with strong associations to CVD and its comorbidities.

### Prioritized targets and drugs

To assess the “novelty” and “potential” of our priority targets, we devised a scoring system inspired by previous methodologies [43,44]. This system encompasses 6 criteria, with the overall score being the aggregate of the criteria met: (a) genes deemed significant from GWAS of CVD, exhibiting OR ≥ 1.05 or ≤ 0.95 and *P* ≤ 0.01; (b) genes derived significant from colocalization analysis of CVD, exhibiting PH4 value > 0.8; (c) genes prioritized through an extensive PubMed literature review; (d) genes identified as significant in CVD or their associated complications; (d) phenotypes linked to CVD or related complications, obtained via gene-based PheWAS with *P* < 5.00 × 10^−8^; and (e) genes annotated as therapeutic targets in databases like DrugBank [15], ChEMBL [16], and The Human Protein Atlas [17]. Targets fulfilling 4 of these criteria were classified as high potential, while those not meeting this benchmark were regarded as relatively novel and less studied. Targeted drugs were selected based on their associations with the identified targets in DrugBank and ChEMBL, prioritizing those with direct target action and clinical development stages: marketed products, phase III trials, phase II trials, phase I trials, and preclinical studies. Consequently, we identified preferred drug targets and potential targeted therapeutic agents.

### Machine learning model for CVD diagnosis

In the present investigation, aimed at assessing the predictive capacity of candidate targets for disease, we derived the relative abundance of each target from serum proteome data encompassing 30 subjects with CVD and an equal number of healthy controls. Utilizing this dataset, we applied the previously documented Extreme Gradient Boosting (XGBoost) machine learning technique for constructing predictive models [45]. The samples were randomly partitioned into training and test subsets at a 0.6 ratio. For parameter tuning, we used the R package “caret” [46], initiating with a grid that encompassed 100 iterations, a depth constraint of 6, a learning rate (η) of 0.1, a minimum loss reduction threshold before node splitting of 0.1, a feature sampling fraction of 80%, a minimum sum of weights for child nodes set to 3, a sampling fraction of 80%, and several other parameters. Upon optimization, we computed various metrics, such as accuracy and precision, for both training and evaluation datasets. ElasticNet regression emerged as the top performer in terms of the area under the curve (AUC) on cross-validated training data, and the receiver operating characteristic (ROC) curve was depicted using the R package “pROC” [47]. The significance of individual proteins within the ElasticNet model was deduced directly from their respective weights, while the SHAP [48] values for pivotal features were visualized with the R package “shapviz”. Furthermore, a confusion matrix was produced with the aid of the R package “ggplot2” [49]. The entire machine learning workflow was executed in RStudio (2024.04.2+764) utilizing R version 4.3.3.

### Statistics

In the present research, statistical comparisons between 2 groups were analyzed using the Wilcoxon Mann–Whitney test, whereas for assessments involving 3 or more groups, the Kruskal–Wallis test was adopted. Statistical significance was determined by setting a *P* value threshold of ≤0.05. A range of graphical representations, encompassing volcano plots, Sankey flow diagrams, scatter plots, violin plots, and heatmaps, were created utilizing R packages like “ggplot2” and “ComplexHeatmap” [50]. The comprehensive set of analytical procedures and graphical production was carried out within RStudio (version 2024.04.2+764), employing R software version 4.3.3.

## Ethical Approval

This research employed solely aggregated datasets, excluding individual participants. The ethical clearance has been documented in the referenced studies.

## Data Availability

The data utilized in this study originated exclusively from publicly accessible databases, as outlined in Table S1. The lead contact can provide any supplementary details necessary for reevaluating the reported data.
